# Ophthalmology

**Published:** 1893-12

**Authors:** F. R. Cross


### OPHTHALMOLOGY.

Since Schœler of Berlin advocated injection of tincture of iodine into the eyeball for detachment of the retina, Bull of New York and other experimenters have reported, as was to be expected, not only many failnres of the method, but cases of serious inflammation and injury to the eye.

De Schweinitz, in a recent lecture[1] on the causes and symptoms of detachment of the retina, quotes a *résumé*, published by Emil Grosz, of the methods of treatment now in use, in which some satisfactory results are quoted from the Ophthalmic Clinic of Professor Schulek in Buda Pesth: (*a*) *Puncture of sclera*, 21 cases; 14 no result, 4 improved, 3 made worse. (*b*) *Iridectomy*, 18 cases; 7 no result, 6 improved, 5 made worse. (*c*) *Puncture of retina*, 2 cases; no result. (*d*) *Pilocarpine injections*, 16 cases; improvement in 6, no result in 10. (*e*) *Iodine injections*, in 2 cases with no result. (*f*) *Combined puncture of sclera and pilocarpine injections*, 9 cases; 6 no result, 3 improvement.

The writer's experience is that all methods (excepting iodine injections, which he dares not try) show temporary improvement; mainly, he believes, on account of the rest ensured to the head and eye. A long rest in bed gives the best prospect of arresting the further development of the detachment, and nearly always results in improvement, which may be only temporary. Pilocarpine is of probable value in addition to rest; and he has seen no harm done from scleral puncture, which in several of his cases has been distinctly beneficial. Special circumstances might indicate that iridectomy would be preferable.

G. de Schweinitz has recently[2] reported the results of some experiments undertaken to test the effect of antiseptic injections on the vitreous body in a healthy or inflamed condition. In almost every case a permanent lesion of the vitreous was produced in healthy rabbits' eyes by the injection of two to five minims of mercuric chloride, mercuric cyanide, aqua chlorinata, trichloride of iodine, or pyoktanin. In four rabbits, purulent hyalitis which had been caused by the introduction of staphylococcus received no benefit from strong antiseptic injections. Some improvement, however, was effected in the eye of a dog

[1] *Ther. Gaz.*, Jan., 1893.

[2] *Journ. Amer. Med. Assoc.*, Oct. 21, 1893.

under similar conditions, and possibly further investigation may show that in the dog the treatment may give better results than in the rabbit.

Schapringer [1] discusses the proximate cause of the transient short-sight that may accompany iritis.

Several theories have been adduced to explain the increase of refraction that occurs.

Mittendorf attributed it to an infiltration of the anterior portion of the vitreous, which thus advanced the lens and with it the posterior principal focus of the eye. Such a displacement, to be effectual, would cause considerable pressure on the suspensory ligament and symptoms of eyeball tension; moreover, exudation in the vitreous would alter the refractive index of this body, and thus modify the general refraction of the eye.

Dr. Oliver suggested that spasmodic action of the ciliary muscle caused the myopia; but the latter state continues despite full atropisation, and active spasm rarely accompanies prolonged inflammation.

Schapringer believes that the transient myopia of iritis is best explained by temporary increase of the refractive index of the aqueous humour. He assumes, with fair reason, that the turbidity of the aqueous and the deposited particles are associated with an excess of fibrin in solution of the humour. The clinical suggestion from this hypothesis is, that atropine should not be used longer than necessary, as, by blocking the filtration angle, it still further impedes the osmosis of the too colloid aqueous.

Dr. George Jacoby [2] quotes two cases of rapid and transitory blindness in pertussis. A boy aged 8, who had suffered two months from whooping-cough, suddenly complained of headache, irritability and vomiting, with absolute blindness. His pupils, the media and fundus of both eyes were normal, and he recovered after a few days, complete right hemianopsia temporarily supervening. A girl aged 6, who also became blind after some weeks of whooping-cough, exhibited double optic neuritis, without any hemorrhages or any symptoms of cerebral disturbance. Pupillary action was paralysed in full dilation. Recovery occurred after a few days. Sudden œdema of the cerebral optic nerve tracts probably accounted for the symptoms. A few cases of ischæmia retinæ, with temporary loss of sight, have been reported during whooping-cough. Typhus, scarlet fever, and other exanthemata have been occasionally complicated by sudden

[1] *N. Y. Med. Journ.*, Oct. 21, 1893. [2] *Ibid*, Feb. 28, 1891.

complete loss of sight. The condition of the pupils and of the eye fundus is usually unaffected, and recovery follows in a few days.

Heredity plays an important *rôle* in diseases of the eye, as well as in those which affect other parts of the body. Thus M. Despagnet [1] recorded that five children of the same parents, the father being an alcoholic subject, were each attacked, at ages varying from 28 to 32, with double optic neuritis and violent headache, possibly associated with meningitis. There was no other cause for the neuritis, which was associated with marked contractions of the visual fields in each case, and resulted in partial atrophy of the nerve. A child of one of the five mentioned also suffered from similar symptoms.

Exophthalmic goitre has been recorded as affecting several members of the same family, and striking instances of the heredity of cataract have been recorded over and over again. Leber and other ophthalmologists have given good proof of the existence of a form of optic atrophy which is transmitted through many members of the same families, and which has been described as hereditary amblyopia, hereditary optic neuritis, or hereditary optic atrophy. The disease usually appears in early adult life, but it has been recorded in children and in patients beyond fifty years of age.

Pilocarpine has been strongly recommended in full doses for rheumatic affections of the eyeball; if larger doses are not well borne by the patient, smaller doses still have a curative influence.[2] Dr. Mansel Sympson [3] publishes an interesting case of recurrent amaurosis without any ophthalmoscopic changes preceding uræmic convulsions, in which subcutaneous injections of pilocarpine proved of much service.

Chibret suggested tincture of iodine as a local application to corneal ulcers. Sedan[4] states that it is a painful application, and that it forms deposits in the *cul de sac* of the conjunctiva as well as over the corneal surface. Bane [5] uses for ulcerative blepharitis a light application of tincture of iodine preceded by a ten per cent. solution of cocaine and followed by an oil.

Dr. Ferret [6] considers that senile cataract may largely depend upon method of alimentation. He suggests that an

[1] *Prog. Méd.*, June 18, 1892.

[2] *Ther. Gaz.*, vol. xvi., p. 602. [3] *Pract.*, Jan., 1893.

[4] *Rec. d'Ophth.*, 1891, xiii., p. 710. [5] *N. Y. Med. Journ.*, Nov. 12, 1892.

[6] *De la Cataracte corticale.*, Paris, 1893.

excessive amount of salt in the food, or some defect of system which leads to the faulty elimination of salt from the tissues, is an important factor in etiology. Cataract, though rare in Algeria, is very common amongst the Arabs employed in the rock-salt works near Djelfa. Dr. Ferret also thinks he has proved its frequency elsewhere in the neighbourhood of salt pits—and he alludes to Kunde's experiments on frogs, which support the same theory.

Not only in reading, but also in writing, a faulty position tends to defective eyesight. Mayer[1] states that an examination of two thousand children in Bavaria shows that, with upright writing, 55 per cent. of the children sat in a good position, while only 5 per cent. sat properly of those whose writing was sloping. Vertical writing has long been recognised to encourage a good position and comfort for the pupil, while the attitude is cramped and unnatural when writing is sloped, as is usually taught in this country.

In order to produce paralysis of the accommodation by means of homatropine, authorities say that repeated instillations of the drug at frequent intervals are necessary. Coleman[2] has suggested that one drop of a neutral solution, one grain to the drachm, should be introduced into the conjunctival sac over-night, and in the morning one drop should be instilled every quarter of an hour for one hour and a half; the eye to be examined not less than forty-five minutes, nor more than sixty minutes, after the last application. Should this method really prove as effectual as that by atropine in thoroughly neutralising spasm of accommodation, it must be far more convenient by reason of the comparatively slight discomfort which accompanies the use of the more transitory mydriatic.

M. Galtier[3]—having noted that Brown-Séquard has reported improvement in cases of locomotor ataxia by injection of organic liquids—recently treated a case of grey optic nerve atrophy by injections of testicular fluid. The central vision was improved from one-tenth to one-sixth, and there was also increase in lateral range and in perception of colours.

F. R. Cross.

[1] *Amer. Pract. and News*, March 11, 1893.

[2] *Ann. Ophth. & Otol.*, 1893, ii., p. 70. [3] *Ann. d'Ocul.*, May, 1893.

